# Helen’s twins in the Balkans: discovery of two new *Paraptychoptera* Tonnoir, 1919 species closely related to *P.helena* Peus, 1958, with systematic revision of the “lacustris” group (Diptera, Ptychopteridae)

**DOI:** 10.3897/zookeys.1071.58598

**Published:** 2021-11-17

**Authors:** Lujza Keresztes, Jürgen Kappert, Mária Henning, Edina Török

**Affiliations:** 1 Hungarian Department of Biology and Ecology, Center of Systems Biology, Biodiversity and Bioresources, Advanced Hydrobiology and Biomonitoring Lab, Babeş-Bolyai University, 400006 Cluj-Napoca, Romania; 2 Forsthaus 1, D-363 94, Sinntal, Germany; 3 “Lendület” Landscape and Conservation Ecology, Institute of Ecology and Botany, Centre for Ecological Research, 2163 Vácrátót, Hungary

**Keywords:** Cladistics analyses, identification key, male genital structures, new species, phantom craneflies, Ptychoptera, TNT phylogeny

## Abstract

*Ptychopteracastor* Keresztes & Kappert, **sp. nov.** and *P.pollux* Keresztes & Török, **sp. nov.** both belong to the subgenus Ptychoptera (Paraptychoptera)Tonnoir (1919) and are described from boggy headwaters in the south Balkan area. These new species are closely related to the range-restricted *P.helena* Peus, 1958, which is known only from Oiti village, Mount Oeta, Phthioitis region, Greece and, together with *P.lacustris*, forms a morphologically well-defined unit in the subgenus Paraptychoptera. Based on cladistic analyses of 53 different morphological characters using the male antenna, wing, and genital structures, a general revision of the “lacustris” group is proposed with a dichotomous key of *Paraptychoptera* species.

## Introduction

Ptychopteridae or phantom crane flies are medium- to large-sized flies with slender shiny black body, sometimes with yellow or reddish markings, and long legs with tipuloid appearance, however they differ by several characters including their having a small membranous lobe at the base of the halter (Stubbs 1993; Andersson 1997). Ptychopteridae are remnants of a small, archaic family of Diptera with less than 85 recent species distributed worldwide, but they are absent from Australasia and Oceana (Fasbender 2014; Eskov and Lukashevich 2015).

Extant representatives are classified in two subfamilies, Ptychopterinae with a single genus, *Ptychoptera* (about 70 species) and Bittacomorphinae with two other genera, *Bittacomorpha* (with only 2 species) and *Bittacomorphella* (8 species) (Fasbender 2014). Only sixteen *Ptychoptera* species are present in Europe (Török et al. 2015), from which the monophyletic western Palaearctic *Paraptychoptera* group was proposed first by Tonnoir (1919), sharing a conspicuous invaginated auxiliary sexual organ on the male abdominal sternite III. Later, the group was synonymised by Alexander (1927) and included between the more widespread *Ptychoptera* and, consequently, was ignored in important revisions and contributions to the European Ptychopteridae (Freeman 1959; Peus 1958; Zitek-Zwyrtek 1971; Deliné-Draskovits 1983; Krzemiński 1986; Rozkosny 1997; Krzemiński and Zwick 1993). *Paraptychoptera* was recovered only recently as a subgenus of *Ptychoptera* (Zwick and Starý 2003; Fasbender 2014) and also referred to by Ujvárosi et al. (2011). Strong morphological and molecular support to monophyly of *Paraptychoptera* were added in a reference contribution of the world Ptychopteridae species (Fasbender 2014). In his milestone work on modern Ptychopteridae research, Fasbender (2014) recovered Ptychoptera (Paraptychoptera)Tonnoir (1919) as a monophyletic group sharing a series of common diagnostic features in male genital structures, such as the poorly sclerotised epandrial claspers, without basal lobes, but variable ventral lobes, the well-developed tongue-like hypoproct, gonostylus with basal lobe divided into knoblike anterior lobe and sickle-like medial lobe, and the presence of a conspicuous invaginated auxiliary sexual organ on sternite III.

*Paraptychoptera* is a monophyletic western Palaearctic group of Ptychopteridae, largely European in distribution, and with only a few species being present in Western Asia and North Africa. Only ten species are considered here, following Fasbender (2014): P. (Paraptychoptera) lacustris Meigen, 1930; *P.longicauda* Tonnoir, 1919; *P.paludosa*, Meigen 1804 has wider European distribution while *P.handlirschi* Czizek, 1919 and *P.silvicola*, Zwyrtek & Rozkosny, 1967 are restricted to Central or Southern Europe. Another five species, however, have a more restricted distribution: P. (P.) agnes Krzemiński & Zwick, 1993 is an endemic species that is described from the Pilis mountains, in the Transdanubian Mountains, Hungary (Krzemiński and Zwick 1993), P. (P.) delmastroi Zwick & Stary, 2003 from Villafranca municipality in the City of Turin, and Cantarana municipality (both are in the region of Piemonte), northern Italy (Zwick and Starý 2003) and Tunisia (Paramonov, 2013) while P. (P.) helena Peus, 1958 is known only from a single site, Oiti village, Mount Oeta, Phthioitis region, Greece (Peus 1958). Also considered, are two extra-European species that belong to the subgenus *P.resseli* Theischinger, 1978 from the vicinity of Noshahr city, Noshahr region, Mazandaran Province, Iran and P. (P.) surcoufi Séguy, 1926 from Algeria (Fasbender 2014).

Among *Paraptychoptera*, the lacustris group was first proposed by Tonnoir (1919), as species having less developed, but recognisable, auxiliary sexual organs in the male sternite III, and was also referred to by Stubbs (1993) and Zwick and Starý (2003), and included the following species: *P.lacustris*, *P.longicauda*, *P.paludosa*. In the present paper we describe two new species of the Ptychoptera (Paraptychoptera) lacustris from the South Balkans, and provide a key of the revised lacustris group, within which the two new species belong. Material of the range-restricted North African *P.surcoufi* were not available throughout the present investigation, and a detailed description of morphological features has not been published since its summary description in Séguy (1926).

## Materials and methods

**Figure 1.**
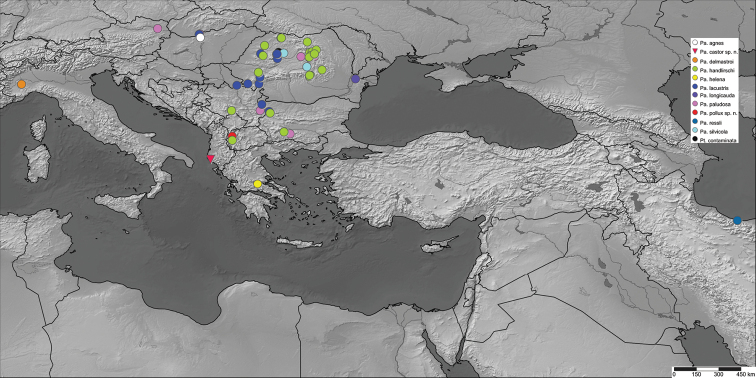
Distribution of different *Paraptychoptera* species used in the present study.

The type material of *Paraptychoptera* that was used in this study was acquired through field collections by the present authors. Seventy (70) male specimens belonging to nine different species originating from different parts of Europe were investigated (Fig. 1, Table 1).

**Table 1.** List of taxa used in this study.

Taxa	Nr. ind	Source of material	Coordinates	Collectors
P. (Pa.) agnes Krzemiński & Zwick, 1993	0	literature data: Krzemiński & Zwick, 1993, Fasbender, 2014	–	–
P. (Pa.) castor sp. nov.	1	Albania, Vlora, Tragjas, Repet y Izvorit, 17 m, 30.iv.2019	40.323132°N, 19.510031°E	leg. Henning, M.
P. (Pa.) delmastroi Zwick and Starý, 2003	0	literature data: Zwick and Starý, 2003 , Fasbender, 2014	–	–
P. (Pa.) handlirschi (Czizek, 1919)	3	Romania, Baia Sprie, Gutin Mts., 955 m, 15.v.2014	47.698860°N, 23.794682°E	leg. Keresztes L.
P. (Pa.) helena Peus, 1958	1	paratype, NMBG Germnay	–	–
P. (Pa.) lacustris Meigen, 1830	1	Bulgaria, Berkovitsa, small brook, 616 m, 28.v.2013	43.218602°N, 23.071343°E	leg. Keresztes L.
	4	Bulgaria, Dabravka, Dabravka lake shore, 364 m, 30.iv.2012	43.651059°N, 22.628539°E	leg. Török E.
	1	Bulgaria, Velingrad, Rhodope Mts., 870 m, 12.vi.2008	41.986014°N, 23.971926°E	leg. Keresztes L.
	7	Hungary, Szobi, Ipolytölgyes, Börzsönyi Mts., 358 m, 2.vi.2016	47.910624°N, 18.821948°E	leg. Török E.
	1	Romania, Cluj, Gilău Mts., Pecica brook, 443 m, 16.iv.2016	46.733137°N, 23.552135°E	leg. Keresztes L.
	3	Romania, Poiana Mărului, Șureanu Mts., 1444 m, 16.vi.2008	45.316562°N, 22.517324°E	leg. Keresztes L.
	1	Romania, Rimetea, Bedeleuilui Mts., 531 m, 28.V.2007	46.448873°N, 23.570063°E	leg. Keresztes L.
	8	Romania, Sasca Română, Almașului Mts., 266 m, 8.v.2009	44.926365°N, 21.782738°E	leg. Keresztes L.
	9	Romania, Valea Iadului, Bihor Mts., Leșu lake, 691 m, 21.v.2006	46.745951°N, 22.556599°E	leg. Keresztes L.
	2	Serbia, Voivodina, Šušara, Deliblatska Peščara, 111 m, 10.vii.2013	44.831943°N, 21.111992°E	leg. Török E.
P. (Pa.) longicauda (Tonnoir, 1919)	3	Romania, Luncavița, Măcin Mts., 151 m, 1.vi.2006	45.221240°N, 28.320892°E	leg. Keresztes L.
P. (Pa.) paludosa Meigen, 1804	1	Austria, Wien, Hermannskoegel, 326 m, 21.v.2013	48.261830°N, 16.302293°E	leg. Graf, W.
	3	Bulgaria, Fotinovo, Rhodope Mts., 1495 m, 16.vii.2012	41.870489°N, 24.344398°E	leg. Keresztes L.
	4	Hungary, Nagybörzsöny, Börzsönyi Mts., 350 m, 1.v.2016	47.939197°N, 18.859785°E	leg. Török E.
	8	Hungary, Szobi, Ipolytölgyes, Börzsönyi Mts., 358 m, 2.vi.2016	47.910624°N, 18.821948°E	leg. Török E.
	2	Romania, Sândominic, Hăghimaș Mts., Babos Laka, 809 m, 9.vi.2019	46.573520°N, 25.822180°E	leg. Keresztes L.
	1	Romania, Voșlobeni, După Luncă peat bog, 757 m, 6.vi.2017	46.670458°N, 25.659906°E	leg. Keresztes L.
	2	Serbia, Fruška Gora National Park, Čerević, 501 m, 5.vii.2018	45.156725°N, 19.738838°E	leg. Keresztes L.
P. (Pa.) pollux sp. nov.	1	North Macedonia, Novo Selo, Mavrovo lake, 990 m, 29.vi.2017	41.721355°N, 20.830103°E	leg. Török E.
P. (Pa.) resseli Theischinger, 1978	0	literature data: Fasbender, 2014	–	–
P. (Pa.) silvicola Zwyrtek & Rozkosny, 1967	3	Romania, Voșlobeni, După Luncă peat bog, 757 m, 6.vi.2006	46.670458°N, 25.659906°E	leg. Keresztes L.
P. (Pa.) surcoufi Séguy, 1926	0	literature data: Fasbender, 2014	–	–
P. (Pt.) contaminata (Linnaeus, 1758)	3	Romania, Florești, marshy area, Someșul Mic, 370 m, 18.v.2019	46.749129°N, 23.476040°E	leg. Keresztes L.
	2	Bulgaria. Primorsko, Ropotamo Nature Reserve, 9 m. 11.vii.2018	42.301909°N, 27.727464°E	leg. Keresztes L.
**Total**	**75**			

Low resolution photos of the whole fly and the wings were taken with a stereomicroscope (Zeiss Stemi 2000-C) and a consumer digital SLR camera (Cannon 1100D). Photos of different genital structure parts were taken with a compound microscope (Motic 310 BA) that was equipped with standard plan-achromatic objectives and additionally with objectives from the inverse microscope of the same manufacturer, for work on glycerol without coverslip. The camera was of the high-resolution USB CCD type (Imaging Source Europe GmbH DFK 51AU02). As stacking software, we used the Hugin suite (SourceForge.net). Male genitalia were left overnight in 10% potassium hydroxide (KOH) and for one hour in undiluted glacial acetic acid, to neutralise and wash out the soap that was created from the soft tissues. The male genitalia were then transferred to a larger amount of glycerol to wash out the acid. Afterwards, they were transferred to a drop of glycerol on a slide with rounded excavation. The genitals were dissected, the parts were oriented using the stereomicroscope, and then the slide was carefully transferred to the compound microscope for the taking of photos. Finally, the parts were washed again in 100% isopropanol and embedded permanently in artificial Canada Balsam (Malinol), whereby high resolution photos were taken. Stacking results in general consist of 5-10 single exposures with the stereomicroscope and of 10-50 exposures with the compound microscope.

The types of *P.castor* sp. nov. and *P.pollux* sp. nov. are deposited in the Diptera collection of the Faculty of Biology and Geology, Babeş-Bolyai University, Cluj-Napoca (UBB), Romania (DCFGB). The study of *P.helena* was based on a paratype male from the Zoological Research Museum Alexander Koenig (*ZFMK*), Bonn, Germany, Museum-Id ZFMK-DIP-00015966.

*Paraptychopteraagnes* Krzemiński & Zwick, 1993, *P.delmastroi* Zwick & Stary, 2003, and *P.ressli* Theischinger, 1978 were not available during the present study, but the detailed morphological description of wing and male terminalia, based on Krzemiński and Zwick (1993) and Zwick and Starý (2003), were used to evaluate discrete details on male terminalia. A comprehensive morphological dataset of the world Ptychopteridae published recently by Fasbender (2014) was also used as a source for morphological details of the male terminalia in species where fresh material were not available to us during the study. Terminology of wing venation and genitalic morphology of *Paraptychoptera* Tonnoir, 1919 follows Fasbender (2014). Cladistic analyses of 53 morphological characters on antennae, wing and male terminalia were analysed (Table 2).

**Table 2.** Morphological characters of male *Paraptychoptera* specimens used in the phylogenetic analyses.

1	Antennal segments: (0), segment 3 equal with segments 4+5; (1) segment 3 shorter than segments 4+5
2	Wing R2+3+4: oblique straight (0); curved or angled in the middle (1)
3	Wing: R2+3+4 length: (0) > 2 × length of rm; (1) < 2 × length of rm
4	Male auxiliary sexual organ on abdominal sternite III: only cluster of setae (0); cluster of setae and distal lobes (1)
5	Male auxiliary sexual organ, distal lobe: absent or weakly developed (0), well developed, ventrally projected outer lip depressed in the middle (1)
6	Male auxiliary sexual organ, distal lobe: outer lip truncate or straight: (0) absent; (1) present
7	Median sclerotized strip of the auxiliary sexual organ with a transverse ornamentation: (0) absent; (1) present
8	Epandrial clasper: well sclerotized with squared basal lobe and complex ventral lobe (0); poorly sclerotized without basal lobe (1)
9	Epandrial clasper configuration: (0), simple cylindrical; (1), cylindrical with additional subterminal ventral lobes
10	Epandrial clasper configuration: (0), simple cylindrical; (1), cylindrical with basal lobes
11	Epandrial clasper apex: rounded (0), pointed and curved (1)
12	Epandrial subapical process: absent (0); present (1)
13	Epandrial subapical process bilobate: absent (0); present (1)
14	Epandrial subapical process bilobate, inferior arm chitinous and curved: (0) absent; (1) present
15	Epandrial subapical process bilobate, superior arm laterally compressed, lobate, inferior arm truncate: (0) absent; (1) present
16	Epandrial subapical process bilobate, both arms chitinous and curved: (0) absent; (1) present
17	Epandrial subapical process bilobate, superior arm beak-shaped, inferior arm triangular: (0) absent; (1) present
18	Epandrial subapical process with chitinous process having curved apex: (0) absent; (1) present
19	Epandrial subapical process with a rounded apex, and strong basal thorn, longer than subapical process: (0) absent; (1) present
20	Epanadrial subapical process with rounded aped and strong basal thorns, shorter than subapical process: (0) absent; (1) present
21	Hypoproct: reduced to paired rectangular plates in subepandrial membrane (0); hypoproct well developed, triangular or tongue like (1)
22	Hypoproct short triangular lobe with rounded apex: (0) absent; (1) present
23	Hypoproct long, tongue like process: (0) absent (1) present
24	Hypoproct long, tongue like process, apex covered with dense hear: (0) absent (1) present
25	Hypoproct long, tongue like process, apex glabrous and bilobate: (0) absent (1) present
26	Hypoproct long, tongue like process, apex pointed, harpoon-shaped: (0) absent (1) present
27	Hypoproct long, tongue like process, apex truncate or slightly depressed: (0) absent (1) present
28	Gonostylus, shape of anterior lobule: (0) scythe-like; (1), lobe-like with rostrum
29	Gonostylus, shape of medial lobule: lobe like (0); scythe-like (1)
30	Gonostylus, apical stylus of gonostylus apex with strong spines: absent (0); present (1)
31	Gonostylus, apical stylus or gonostylus: simple (0); inflated (1)
32	Gonostylus, secondary lobe of apical stylus: present (0); absent (1)
33	Hypandrium apex terminal division spade like, without trichoid sensilla: (0) present; (1) absent;
34	Hypandrium apex terminal division wide spade like, with trichoid sensilla (0) absent; (1) present
35	Hypandrium apex terminal division long lobe like process, with rounded apex: (0) absent; (1) present
36	Hypandrium apex terminal division long lobe like process with bilobate apex: (0) absent; (1) present
37	Hypandrium eversible sac extended anteriorly nearly to margin: (0), absent; (1), present
38	Hypandrium basal scale: (0), absent; (1) present
39	Hypandrium basal scale chitinous, hat shape: (0), absent; (1) present
40	Hypandrium basal scale chitinous, hat shape, with medial lobe (0), absent; (1) present
41	hypandrium: basal scale lobe like, well developed (0), absent; (1) present
42	Hypandrium basal scale lobe like, weakly developed (0), absent; (1) present
43	Aedeagus: ejaculatory apodeme size: (0), larger, than sperm sac; (1), subequal
44	Aedeagus subapical plate wide spatulate: (0) present; (1) absent
45	Aedeagus subapical plate narrow, apex rounded or pointed: (0) present; (1) absent
46	Aedeagus subapical plate narrow, apex depressed: (0) present; (1) absent
47	Paramere apical lobes filiform process: (0) present; (1) absent
48	Paramere apical lobes with a notch in the middle: (0) present; (1) absent
49	Paramere apical lobes tip pointed: (0) present; (1) absent
50	Paramere apical lobes tip rounded: (0) present; (1) absent
51	Paramere lateral arms reduced: (0) present; (1) absent
52	Paramere lateral arms long, beak shaped: (0) present; (1) absent
53	Paramere lateral arms short, truncate: (0) present; (1) absent

Morphological characters were selected based on the world phylogenetic revision of Ptychopteridae (Fasbender, 2014), but completed with new morphological data. Ptychoptera (Ptychoptera) contaminata was considered as an outgroup taxon. Missing data were coded as ‘?’. The list of morphological characters is presented in Table 3.

**Table 3.** Matrix of the 53 morphological items of data used in the phylogenetic analyses. For the description of characters and character states see text. Missing data were coded as ‘?’.

	1	2	3	4	5	6	7	8	9	10	11	12	13	14	15	16	17	18	19	20	21	22	23	24	25	26	
***contaminata***	0	0	0	0	0	0	0	0	0	0	0	0	0	0	0	0	0	0	0	0	0	0	0	0	0	0	
*agnes*	?	1	1	1	?	?	?	1	1	0	0	1	1	1	0	0	0	0	0	0	1	0	1	1	0	0	
*castor* sp. nov.	1	1	1	1	0	1	0	1	0	0	0	1	0	0	0	0	0	0	0	1	1	0	1	0	0	1	
*delmastroi*	?	?	?	?1	1	0	1	1	0	1	0	1	0	0	0	0	0	1	0	0	1	1	0	0	0	0	
*handlirschi*	1	1	1	1	1	0	1	1	0	1	0	1	1	0	1	0	0	0	0	0	1	1	0	0	0	0	
*helena*	1	1	1	1	0	1	0	1	0	0	0	1	0	0	0	0	0	0	1	0	1	0	1	0	1	0	
*lacustris*	1	1	1	1	0	1	0	1	0	0	0	1	0	0	0	0	0	0	0	0	1	1	0	0	0	0	
*longicauda*	1	1	1	1	1	0	1	1	0	1	0	1	0	0	0	0	0	1	0	0	1	1	0	0	0	0	
*paludosa*	1	1	1	1	1	0	1	1	0	1	0	1	1	0	0	1	0	0	0	0	1	1	0	0	0	0	
*pollux* sp. nov.	1	1	1	1	0	1	0	1	0	0	0	1	0	0	0	0	0	0	1	0	1	0	1	0	0	0	
*resseli*	?	?	?	?	0	?	?	1	0	0	1	1	0	0	0	0	0	1	0	0	1	0	1	1	0	0	
*silvicola*	1	1	1	1	1	0	1	1	0	1	0	1	1	0	0	0	1	0	0	0	1	1	0	0	0	0	

	**27**	**28**	**29**	**30**	**31**	**32**	**33**	**34**	**35**	**36**	**37**	**38**	**39**	**40**	**41**	**42**	**43**	**44**	**45**	**46**	**47**	**48**	**49**	**50**	**51**	**52**	**53**
***contaminata***	0	0	0	0	0	0	0	0	0	0	0	0	0	0	0	0	0	0	0	0	0	0	0	0	0	0	0
*agnes*	0	1	1	0	1	0	1	1	0	0	1	1	0	0	0	1	1	1	1	0	1	1	0	0	1	0	1
*castor* sp. nov.	0	1	1	1	0	0	1	0	1	0	1	0	0	0	1	1	1	1	0	1	1	0	1	0	1	0	1
*delmastroi*	0	1	1	0	0	0	1	1	0	0	0	0	1	0	0	1	1	1	1	0	1	1	0	0	1	1	0
*handlirschi*	0	1	1	0	0	0	1	1	0	0	0	0	1	0	0	1	1	1	1	0	1	1	0	0	1	1	0
*helena*	0	1	1	1	0	0	1	0	1	0	1	0	0	0	1	1	1	1	0	1	1	0	1	0	1	0	1
*lacustris*	0	1	1	0	0	0	1	0	1	0	1	0	0	1	0	1	1	1	1	0	1	0	1	0	1	0	1
*longicauda*	0	1	1	0	1	0	1	0	0	1	1	1	0	0	0	1	1	1	1	0	1	0	1	0	1	1	0
*paludosa*	0	1	1	0	0	1	1	1	0	0	0	0	1	0	0	1	1	1	1	0	1	1	0	0	1	1	0
*pollux* sp. nov.	1	1	1	1	0	0	1	0	1	0	1	0	0	0	1	1	1	1	0	1	1	0	1	0	1	0	1
*resseli*	0	1	1	0	0	0	1	1	0	0	0	0	0	1	0	1	1	1	1	0	1	0	0	1	1	1	0
*silvicola*	0	1	1	0	0	0	1	1	0	0	0	0	1	0	0	1	1	1	1	0	1	1	0	0	1	1	0

The morphological data matrix was managed with Mesquite 3.5 (Maddison and Maddison 2019). Maximum parsimony analysis of the morphological data was performed using a parsimony programme: “Tree Analysis using New Technologies” (TNT) version 1.5 (Goloboff and Catalano 2016). A “traditional” search based on 1000 replicates of Wagner trees, through ‘tree bisection reconnection’ (TBR) branch swapping holding 100 trees by the collapsing rule: ‘min. length=0’. Subsequently, we selected the best tree, in terms of species topology and population phylogeographical clades, and resampled with 10000 replicates using a standard bootstrap procedure (Felsenstein 1985). Values at nodes represented absolute frequencies and frequency differences (GC, Group present/Contradicted).

## Results

### Taxonomic account

#### Ptychoptera (Paraptychoptera) castor

Taxon classificationAnimaliaDipteraPtychopteridae

Keresztes & Kappert sp. nov.

922FD119-F93D-561C-898E-8F20FE3A603D

http://zoobank.org/D43DA29E-1941-4B33-BBE9-A3D88D80F277

Figure 2

##### Type material.

***Holotype***. male, Albania: Tragjas municipality, Rrepet e Izvorit, Vlora district, sweeping the vegetation near a limnocren karst spring with large basin and muddy shore with reeds, 30.iv, 2019, 17 m, leg. M. Henning, 40.323132°N, 19.510031°E. Institutional id for specimen is DCFBG-PT-0002.

##### Diagnosis

**Figure 2.**
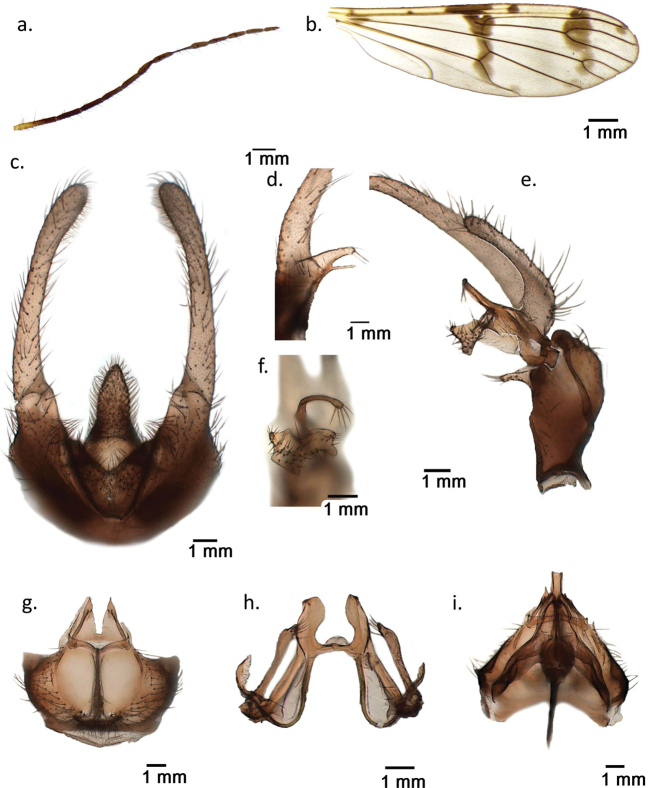
*Ptychopteracastor* sp. nov. **a** flagellum of antennae **b** right wing **c** epandrium, dorsal **d** subapical lobe of epandrium, ventral **e** left gonocoxite with gonocoxite lobes **f** anterior and medial lobules, details **g** hypandrium, caudal **h** paramere, ventral **i** aedeagal complex, dorsal.

**Figure 3.**
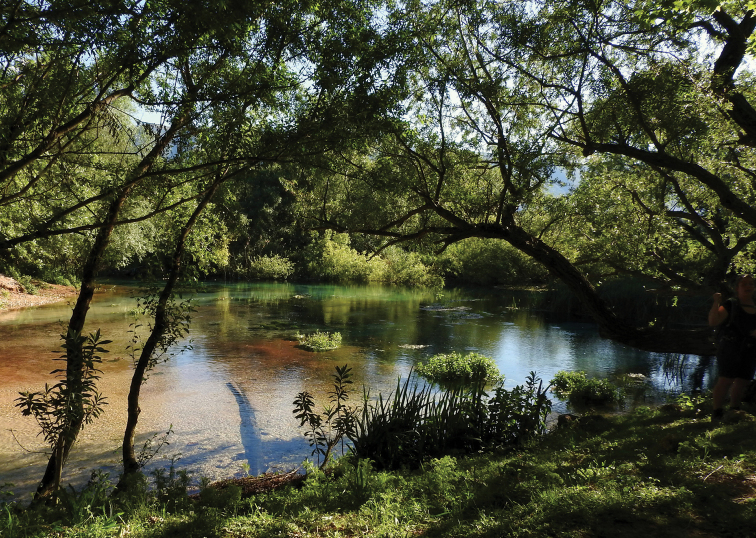
Habitat of *Ptychopteracastor* sp. nov., south-western Albania, Tragjas, Repet y Izvorit.

**Figure 4.**
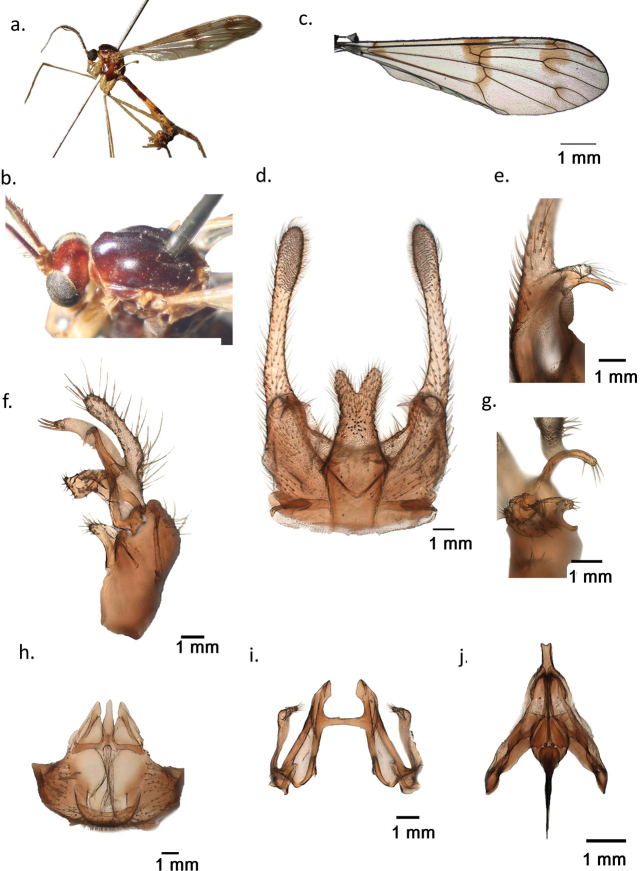
*Ptychopterahelena*, paratype male (ZFMK) **a** habitus male **b** head and thorax dorsal **c** right wing **d** epandrium, dorsal **e** subapical lobe of epandrium, ventral **f** gonocoxite and gonostylus complex, dorsal **g** gonostylus anterior and medial lobules, caudal **h** hypandrium, caudal **i** paramere, ventral **j** aedeagal complex, dorsal.

. Ptychoptera (Paraptychoptera) castor sp. nov. is known only from a single male collected near a limnocrene karst spring with muddy shore invaded by rich vegetation at Repet y Izvorit, Tragjas, Albania (Fig. 3). Male general habitus, wing venation and spots are highly similar to *P.helena* (Fig. 4a, b, c). However, the male epandrium has a unique design, differentiated from all other members of *Paraptychoptera*, but close to *P.helena* (Fig. 4d, e). In contrast to *P.helena*, the finger-like subapical process on its ventral side is well developed, with a basal chitinous process, equal in length with subapical lobe, which is much shorter in *P.helena* (Fig. 2d) and the conspicuous long harpoon-shaped apex of the hypoproct (Fig. 2c) which is bilobate in *P.helena* (Fig. 4d). Gonostylus apical stylus is long, twice as long as the secondary lobe (Fig. 2e), which differentiates it well from *P.helena*, where such a process is subequal. Gonostylus anterior lobule with a short finger-like vental process (Fig. 2f), while in *P.helena* such a process is much longer and curved at tip (Fig. 4g). Hypandrium apex lacking a narrow-lobe-like terminal division (Fig. 2g) which is present in *P.helena* (Fig. 4h), and well developed in all other *Paraptychoptera* species, in addition with a series of fine differences in male aedeagal complex and paramere (Fig. 2h, i).

##### Description

. Medium-sized species, body length 7.3 mm, wing length 8 mm. Head and thorax shiny black, almost glabrous, pleuron almost uniformly brownish, some obvious pale setae only above halter. Head shiny brownish, labrum pale brownish to yellow. Antennae with 15 segments. Scape elongate cylinder, pedicel globular, yellowish, as the half of the first flagellar segment. Remainder flagellomeres blackish brown (Fig. 2a). First flagellar segment shorter than the following two segments together, the others successively shorter and thinner. Each flagellar segment with several long straight black setae and dense pelt of short dark hairs. Eye large, finely faceted, bare; no ocelli. Large, oval, clypeus, convex, terminal labrum yellowish. Large labellum, very long maxillary palpus with whip-like fifth segment pale yellow.

Thorax dorsally black with metallic blue shining, narrow pronotum, base of postnotum and large parts of episternum, epimeron, and metapleuron pale brownish. Coxae and legs yellowish, apex of femur, narrow base and apex of tibia, tarsal segments brownish. Wing with three transverse bands of well-developed confluent dark spots close to anterior margin on basal, middle and distal part of otherwise clear or pale yellowish membrane. Additionally, isolated dark spots are present on both sides of the middle dark band at the level of Sc and at the distal end of R3 (Fig. 2b). Wing membrane with macrotrichia. Prehalter and halter pale yellow.

First abdominal tergite blackish to dark brown with metallic shining, only a narrow yellow stripe near the distal, tergite 2 large part yellowish with brown spot in the middle, distal part shiny black, tergite 3 brownish, tergite 4 and all distal tergites brownish black. Genitalia pale brown. Narrow sternites pale brown at base, becoming yellowish towards the auxiliary sexual organ on segment III. Sternites 4-7 medially reduced to a narrow band with a deep notch in the middle at proximal margins.

Auxiliary sexual organ less developed than in the other members of *Paraptychoptera*, excepting *P.lacustris* and *P.helena*. Sternite 3 with thin long golden hair fringes on sides; its bare middle part lacking the transversally sculptured median sclerite, but distal brownish patch is present at distal end, close to the deep pouch of the auxiliary sexual organ. Distally sternite 3 with deep pouch of the auxiliary sexual organ. Two caudal lips of the pouch less developed, one smooth lateral lobe on each side, covered with dense hair fringe in their interior part. Lateral lips separated by a deep furrow leading to a small oval sclerite inside the pouch and two lateral lobes covered with fine sculptures.

Male terminalia. Epandrium with distinct collar, deeply and widely emarginated behind, hypoproct long lobe-like and densely hairy (Fig. 2c). Hypoproct lobe harpoon shaped, tapering at apex. Epandrium lobes long, slightly widened apically. Subapical process of epandrium with a finger-like ventral projection with basal thorn equal in length with the digitiform process (Fig. 2d). Apex of subapical lobe with long macrotrichia (Fig. 2d). Gonocoxite simple, with its medial appendage as a simple curved pilose lobe. Gonostylus apical lobe short, finger-like, fringe of setae at apex, secondary lobe similar shape, but twice as long as the apical lobe (Fig. 2e). Gonostylus anterior lobules divided into a dorsal triangular process and a ventral part with a short finger-like rostrum. Middle lobe strong sclerotised sickle-like rounded at apex, long fine hairs at tip (Fig. 2f). Hypandrium wide, hemispherical, long, elongate crests in the middle, including a narrow slit between them, from which the eversible sac protrudes. The transverse scale at the base of the slit is less developed, tongue-like, rounded apically, membranous and densely pilose. Hypandrium apex terminal division process missing (Fig. 2g). Aedeagal complex highly similar to other *Paraptychoptera* species. Paramere lateral arms well developed, widened towards a sloping apex. Well-developed setae close to the apex of the paramere arms. Apical processes of paramere well developed, rounded, with a recurring thorn-like formation (Fig. 2h). Apex of aedeagus blunt, depressed medially, subapical lobe of aedeagus pointed (Fig. 2i).

Female unknown.

##### Etymology

. The specific epithet is named after Castor, a god from Greek mythology, the twin brother of Helena, because of its close morphological similarity with *P.helena*.

#### Ptychoptera (Paraptychoptera) pollux

Taxon classificationAnimaliaDipteraPtychopteridae

Keresztes & Török sp. nov.

645AD891-E989-599F-ADBE-40F2164C712B

http://zoobank.org/59BAE5BD-BB77-4D24-AB52-A9AB130FD5B9

Figure 5

##### Type material.

***Holotype***. male, North Macedonia: Mavrovo, Novo Selo, sweeping the vegetation near a small outflow from Mavrovo Lake, 29. vi, 2017, 990 m, leg. E. Török, 41.721355°N, 20.830103°E. Institutional id for specimen is DCFBG-PT-0003.

##### Diagnosis

**Figure 5.**
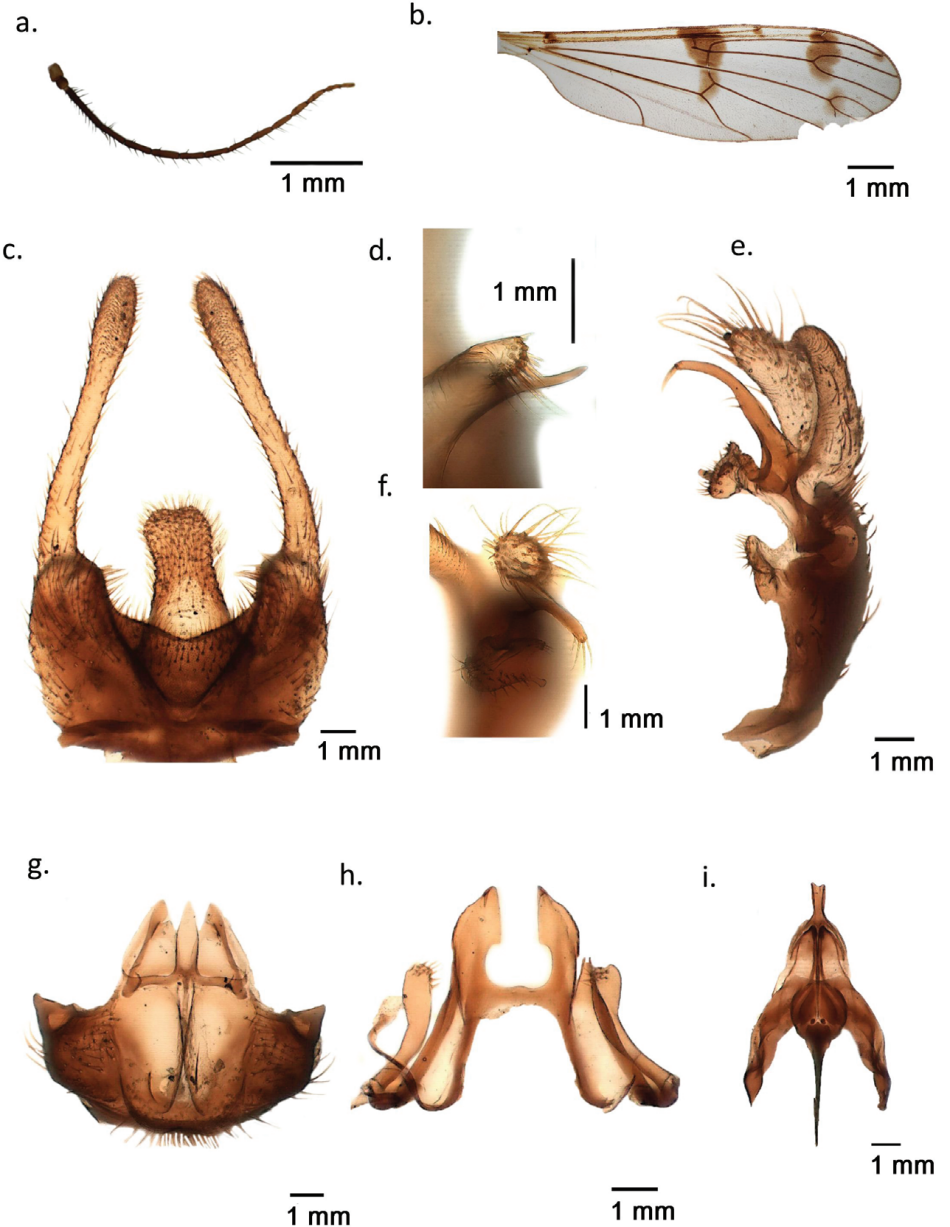
*Ptychopterapollux* sp. nov. **a** flagellum of antennae **b** right wing **c** epandrium, dorsal **d** subapical lobe of epandrium, ventral **e** gonocoxite and gonostylus complex, dorsal **f** gonostylus anterior and medial lobules, caudal **g** hypandrium, caudal **h** paramere, ventral **i** aedeagal complex, dorsal.

**Figure 6.**
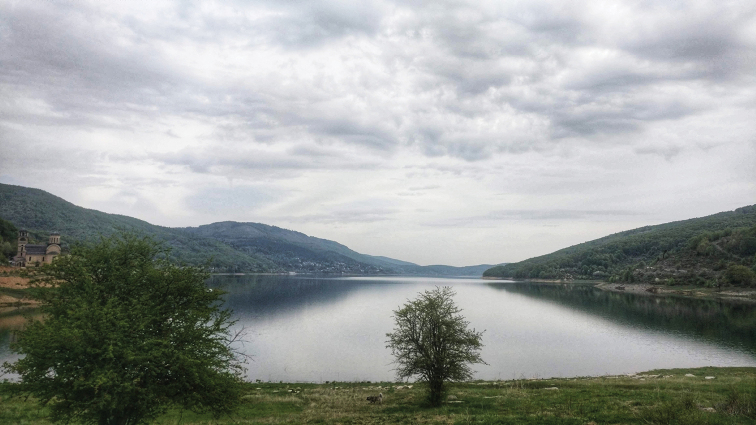
Habitat of *Ptychopterapollux* sp. nov., north Macedonia, Novo Selo village, Mavrovo lake outflow.

. *Paraptychopterapollux* sp. nov. is known only from a single male collected near a small overflow of the Mavrovo Lake with muddy shore invaded by rich vegetation (Fig. 6). The male general habitus and wing venation with spots are highly similar to *P.helena* and *P.castor* sp. nov., but the wing spots tend to be reduced, mostly the basal spot and distally band which is divided into two distinct patches. Further differences are in male epandrium: The less developed finger-like subapical process is unique to *P.helena*, *P.castor* sp. nov., and *P.pollux* sp. nov. (Figs 2d, 4e, 5d). However, in *P.pollux* sp. nov., this process is much shorter than in *P.castor*, but comparably longer than in *P.helena*, with blunt apex and divergent from the basal thorn. However, there is an important difference in the basal thorn orientation. In *P.pollux*, the basal thorn is oriented upward, while in *P.helena* the thorn is curved downward. Hypopygium shape with its rounded and slightly inflated apex is the second most distinctive character of *P.pollux* sp. nov., which differentiates it from both *P.castor* and *P.helena*, as well as from other *Paraptychoptera* species (Figs 2c, 4d, 5c). Paramere lateral arms of *P.pollux* are similar to *P.castor* sp. nov. and *P.helena*, but are shorter and gradually widened at tip and rounded (Fig. 5h). The rest of the characters, such the gonocoxite and gonostylus complex, hypandrium and the aedeagus are highly similar to *P.helena* (Fig. 4g, i).

##### Description

. Medium-sized species, highly similar to its sibling species, *P.castor* sp. nov. and *P.helena*. Body length 7.9 mm, wing length 8.5 mm. Head and thorax similar to *P.castor*. Antennae with 15 segments. Scape elongate, cylindrical, yellowish brown, pedicel globular, pale brown, flagellar segments uniformly dark brown (Fig. 5a). First flagellar segment shorter than the following two segments together, the others successively shorter and thinner. Each flagellar segment with several long straight black setae and a dense pelt of short dark hairs. Head shining black. Eye large, finely faceted, bare; no ocelli. Clypeus large, elongate, rectangular, flattened, labrum pale brownish. Labellum large, yellowish, maxillary palpus very long with a whip-like fifth segment pale yellow.

Thorax dorsally brownish black with metallic blue shining, narrow pronotum, base of postnotum and large parts of episternum, epimeron and metapleuron pale brownish. Coxae orange, legs yellowish, apex of femur, narrow base and apex of tibia, tarsal segments brownish. Wing with three transverse bands of well-developed confluent dark spots in the anterior part of base, middle and distal part of otherwise clear or pale yellowish membrane. Basal spot of the wing more reduced. Distal band interrupted close to ventral edge. Isolated dark spots are present at distal end of Sc and R3 (Fig. 5b). Wing membrane with macrotrichia. Halter and prehalter yellow.

First abdominal tergite blackish to dark brown with a metallic blue shining, narrow yellow stripe close to distal end, well developed yellow band in tergite 2 with a black spot in the middle. Tergite 3 black, covered with yellow setae, the remaining tergites brownish black. Genitalia pale brown. Narrow sternites pale brown at base, becoming yellowish towards the auxiliary sexual organ on segment III. Sternites 4-7 medially reduced to a narrow band with a deep notch in the middle at proximal margins.

Auxiliary sexual organ highly similar to *P.castor* sp. nov. and *P.helena*. Male terminalia. Epandrium with distinct collar, deeply and widely emarginated behind, hypoproct long tongue-like and furry (Fig. 5c). Hypoproct lobe widened at apex, rounded, with a shallow notch in the middle. Epandrium lobe long, slightly widened apically. Subapical process of epandrium with a digitiform ventral projection with basal thorn and curved upward, longer, than the blunt digitiform process (Fig. 5c, d). Apex of the digitiform process with long macrotrichia. Gonocoxite simple, cylindrical. Gonostylus with an anterior lobule divided into a dorsal triangular process, ventral part with a small rostrum (Fig. 5f). Middle lobe strong sclerotised sickle-like curved process, with long fine hairs at the end (Fig. 5e, f). Gonostylus apical lobe narrow fleshly process, subequal with secondary lobe (Fig. 5e). Secondary lobe slightly curved at apex, with strong erect spines. Hypandrium wide, long and elongate crests in the middle, including a narrow slit between them, from which the eversible sac protrudes. The transverse scale at the base of the slit is less developed, with two lateral wings and a triangular process in the middle, membranous and densely pilose. Hypandrium apex terminal division less developed, but distinct as a narrow band with pointed apex (Fig. 5g). Paramere lateral arms less developed, rounded apically and widened with less-developed setae close to the interior of the apex. Apical processes of paramere well developed, rectangular, with a recurring thorn-like formation (Fig. 5h). Aedeagal complex highly similar to other *Paraptychoptera* species. Apex of aedeagus concave, with a depression in the middle, subapical lobe of aedeagus rounded (Fig. 5i).

Female unknown.

##### Etymology

**Figure 7.**
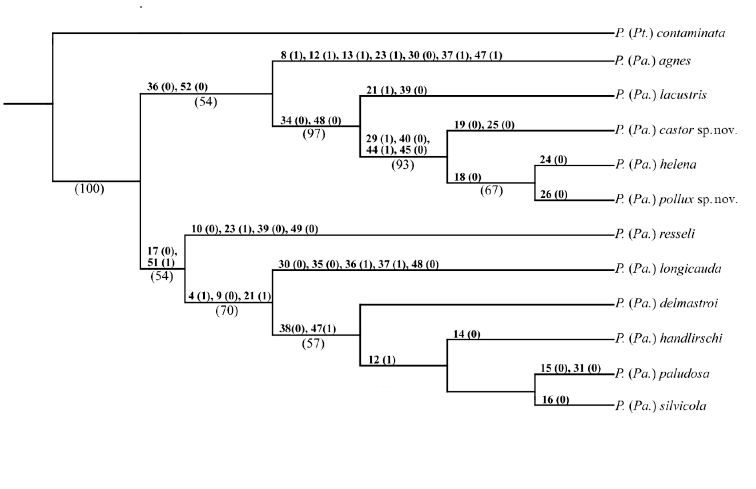
Single most parsimonious tree (1392 steps) based on 53 morphological characters. Bootstrap (B) values over 50% are noted above the corresponding branches, respectively. Branch support was calculated by bootstrap with 10000 replicates. Character states are shown above branches.

. The specific epithet is named after Pollux, the twin brother of Castor in Greek mythology, known together as the Dioscuri, both twin brothers of Helena, because together with *P.castor* they share close morphological similarity with *P.helena* and all together they form a distinct monophyletic unit among *Paraptychoptera*, as was recovered by our cladistic analysis (Fig. 7).

##### Cladistic analyses

.The parsimony analyses of the 53 different morphological characters selected in the present study resulted in a single most parsimonious tree (Fig. 7).

As shown in our parsimony analyses (Fig. 7.), the eleven different *Paraptychoptera* species are divided into two major monophyletic clades, with a highly divergent monophyletic unit, including five species, *P.agnes*, *P.lacustris*, *P.helena*, *P.castor* sp. nov., and *P.pollux* sp. nov. This monophyletic unit is supported by common morphological features, such as the soft and lobe-like basal scale, hypandrium with a long, but not bilobate narrow ribbon-like process or reduced and the simpler lateral arms of paramere (characters 36, 52).

Among this group, a distinct lineage is represented by *P.agnes*, highly different from all other members of the clade by the presence of a conspicuous epandrial lobe, with a ventral lobe close to the base, and a transverse projection with a comb of long setae, unique only to this species, in addition to the particularly shaped hypoproct and epandrial subapical process, and also by the uniquely shaped secondary lobe of the apical stylus inflated and globulose at apex, besides the details of hypandrium and parameres (characters 8, 12, 13, 23, 30, 37, 47).

The present cladistic analyses recovered the “lacustris” species group as a monophyletic unit, which was also noted by Stubbs (1993) and Zwick and Starý (2003), but in a restricted sense, containing only four species: *P.lacustris*, *P.helena*, *P.castor* sp. nov. *and P.pollux* sp. nov. Further, the previously considered *P.loncicauda* and *P.paludosa* were excluded and mostly based on a weekly developed auxiliary sexual organ in “lacustris” group, but well developed in later species, and a weakly developed and reduced subhemispherical membranous basal scale in “lacustris” group in contrast with the well-developed and chitinous scale in *P.longicauda* and *P.lacustris*. There was also the presence of the stripe-like and bifurcate or cross-shaped terminal division of the hypandrium apex in *P.longicauda and P.paludosa*, in contrast with the weakly developed terminal division of hypandrium apex in “lacustris” group. This latter was missing in *P.lacustris* or reduced to a tapering ribbon-like protrusion in *P.helena* and allies (characters 38, 48). However, within the “lacustris” group the subapical lobes of epandrium have a conspicuously similar shape in *P.castor* sp. nov., *P.helena* and *P.pollux* sp. nov., while in *P.lacustris* such a formation is totally absent. *Ptychopteralacustris* is also divergent from the three closely-related Balkan species by the presence of a small triangular hypoproct and rounded subspherical basal scale of the hypandrium (characters 21, 39). *Ptychopterahelena* and the two newly discovered species of the “lacustris” group are morphologically highly similar, but deeply divergent from all other *Paraptychoptera* species, having unique epandrial clasper lobes that are slightly divergent toward the tip, and epandrial subapical lobes reduced to a finger-like short projection with a basal chitinous thorn, hypandrium apex terminal division that is highly reduced, finger-like, with pointed apex, lacking laterally directed spines, but thin hairs are sometimes present (only in *P.castor* such a process is absent). Further, the aedeagus tip has a unique shape, with a small depression in the middle (characters 29, 40, 44, 45). *Ptychopterahelena* and *P.pollux* sp. nov. are highly similar, but minor differences are present in the shape of the hypopygium, and the design of the subapical lobe of the epandrium (characters 24, 26). Further, they are distinctly different from *P.castor* sp. nov. by the presence of a long subapical epandrial lobe (character 19), which is as long as its basal chitinous thorn, as well as the long harpoon-like hypoproct, unique only to this species (character 25).

## Discussion

According to Fasbender (2014), and also supported by the cladistic analyses of the present work, the following morphological diagnostic characters are important to discriminate the *Paraptychoptera* species from all other Ptychopteridae: the presence of well-articulated, but poorly sclerotised epandrial claspers, the apical stylus of gonostylus mostly membranous, with a secondary lobe present, hypandrium well developed, sub-hemispherical, an eversible sac is present, parameres and gonocoxal lobes fused to a supra-aedeagal membrane, paramere lateral arms well developed, and aedeagus having an elliptical ejaculatory apodeme. Within *Paraptychoptera*, the presence of a well differentiated morphologically divergent “lacustris” group was recovered by our cladistic analyses, but excluded *P.longicauda* and *P.paludosa* which were considered in this work based on data in the literature (Freeman 1959; Stubbs 1993; Zwick and Starý 2003). Based on our morphological analyses, the two new species that are described in the present study, *P.castor* sp. nov. and *P.pollux* sp. nov., both belong to the “lacustris” group, and together with *P.helena* they form a well differentiated, range-restricted South Balkan clade, that is highly distant from the more widespread *P.lacustris* and this highlights the importance of the Balkans as an important refuge and genetic hotspot for *Paraptychoptera* in Europe.

A key to the *Paraptychoptera* species was recently provided by Fasbender in 2014 in his global revision of the world Ptychopteridae, including only four *Paraptychoptera* species, instead of the twelve currently known from the Western Palaearctic area. The present key is mostly based on his comprehensive morphology data, but incorporates additional morphological details from the remainder of the *Paraptychoptera* species, including the two newly described species, *P.castor* sp. nov. and *P.pollux* sp. nov. The North African *P.surcoufi* is not included the key because no material was available for the current investigation, nor was there any detailed information in the literature to the best of our knowledge, but its distinctness from the *P.helena* and related species is obvious according to Peus (1958).

### Key to *Paraptychoptera* species (males)

**Table:** 

1	Apical stylus of gonostylus reduced	***P.ressli* Theischinger, 1978**
–	Apical stylus of gonostylus well developed	**2**
2	Epandrial clasper lacking lateral swelling, basal division of hypandrium with a weakly developed membranous basal scale	**3**
–	Epandrial clasper with lateral swelling (mostly reduced in *P.longicauda*), basal division of hypandrium with a well-developed, chitinous projection of different shapes	**7**
3	Epandrial clasper lobes complex, with a transverse projection with a comb of long setae at apex and the presence of a subterminal ventral extension, secondary lobe of the apical stylus expanded into a large balloon-shaped structure	***P.agnes* Krzemiński & Zwick, 1993**
–	Epandrial lobes simple, apical stylus simple, with apex tapering and rounded	**4**
4	Subapical sclerite present at base of epandrial clasper, hypoproct long, tongue-like	**5**
–	Subapical sclerite absent, hypoproct short, triangular	***P.lacustris* Meigen, 1930**
5	Hypoproct furcate apically	***P.helena* Peus, 1958**
–	Hypoproct not furcate apically	**6**
6	Hypoproct apex rounded, with a shallow depression in the middle, apical stylus and secondary lobe subequal in length	***P.pollux* Keresztes & Török, sp. nov.**
–	Hypoproct apex tapered with pointed apex, apical stylus twice as long as the secondary lobe	***P.castor* Keresztes & Kappert, sp. nov.**
7	Epandrial clasper lobe very long, more than twice as long as epandrium length. Apical stylus of gonostylus pendulant, apex of terminal division of hypandrium elongate ribbon-like projection with bifurcate tip, basal scale of hypandrium with no medial triangular projection	***P.longicauda* Tonnoir, 1919**
–	Epandrial clasper lobe short, shorten than epandrium length. Apical stylus of gonostylus not pendulant, apex of terminal division of hypandrium widened, spatulate, basal scale of hypandrium with medial triangular projection	**8**
8	Secondary lobe of apical stylus absent	***P.paludosa* Meigen 1804**
–	Secondary lobe of apical stylus present	**9**
9	Subapical sclerite hook-like	***P.delmastroi* Zwick & Stary, 2003**
–	Subapical sclerite with a paddle-like ventrally projected division	**10**
10	Subapical sclerite with the paddle-like ventrally projected division inflated at apex. With a short hook subterminally and dorsally projected division triangular	***P.silvicola* Zwyrtek & Rozkosny, 1967**
–	Subapical sclerite with ventrally projected arms paddle-like, rounded at apex, without subterminal hooks, dorsally projected division rectangular	***P.handlirschi* Czizek, 1919**


## Supplementary Material

XML Treatment for Ptychoptera (Paraptychoptera) castor

XML Treatment for Ptychoptera (Paraptychoptera) pollux
